# RETRACTED: Kola-Mustapha et al. Network Pharmacology and Molecular Modeling to Elucidate the Potential Mechanism of Neem Oil against *Acne vulgaris*. *Molecules* 2023, *28*, 2849

**DOI:** 10.3390/molecules30234564

**Published:** 2025-11-27

**Authors:** Adeola Tawakalitu Kola-Mustapha, Muhabat Adeola Raji, Oluwakorede Adedeji, George Oche Ambrose

**Affiliations:** 1 College of Pharmacy, Alfaisal University Riyadh, Riyadh 11461, Saudi Arabia; atkmusty@yahoo.com; 2 Department of Pharmaceutics and Industrial Pharmacy, Faculty of Pharmaceutical Sciences, University of Ilorin, Ilorin 240101, Nigeria; 3 Department of Microbiology & Immunology, Alfaisal University, Riyadh 11461, Saudi Arabia; mraji@alfaisal.edu; 4 University of Ilorin Teaching Hospital, Ilorin 240101, Nigeria

The Journal retracts the article titled “Network Pharmacology and Molecular Modeling to Elucidate the Potential Mechanism of Neem Oil against *Acne vulgaris*” [1], cited above.

Following publication, concerns were brought to the attention of the Editorial Office regarding major errors in compound identification [1]. Adhering to our standard procedure, an investigation was conducted by the Editorial Office and Editorial Board, which included an evaluation of the paper’s scientific content and findings. Despite full cooperation from the authors and their explanation that the errors arose from specific analytical issues in the dataset, the Editorial Board could not verify the reliability of the main results and conclusions based on the submitted materials and analysis.

As a result, the Editorial Board decided to retract this article as per MDPI’s retraction policy (https://www.mdpi.com/ethics#_bookmark30).

This retraction was approved by the Editor-in-Chief of the Journal *Molecules*.

The authors agreed on this decision.

## References

[1] Kola-Mustapha A.T., Raji M.A., Adedeji O., Ambrose G.O. (2023). RETRACTED: Network Pharmacology and Molecular Modeling to Elucidate the Potential Mechanism of Neem Oil against *Acne vulgaris*. Molecules.

